# Design and Characterization of Silane-Modified Bio-Based Non-Isocyanate Polyurethane Coatings for Advanced Surface Applications

**DOI:** 10.3390/ma18245551

**Published:** 2025-12-10

**Authors:** Rutu Patel, Ajay Kumar, Mayankkumar L. Chaudhary, Ram K. Gupta

**Affiliations:** 1 National Institute for Materials Advancement, Pittsburg State University, 1204 Research Road, Pittsburg, KS 66762, USA; fajaykumar@gus.pittstate.edu; 2 Department of Physics, Pittsburg State University, 1701 S Broadway St., Pittsburg, KS 66762, USA; 3 Department of Chemistry, Pittsburg State University, 1701 S Broadway St., Pittsburg, KS 66762, USA

**Keywords:** non-isocyanate polyurethane, silane modification, carbonated soybean oil, β-hydroxyurethane–siloxane network, bio-based coating

## Abstract

Non-isocyanate polyurethanes (NIPUs) represent a sustainable alternative to conventional isocyanate-based systems, eliminating toxic reagents while maintaining good performance. In this study, bio-based NIPU coatings were synthesized from carbonated soybean oil (CSBO) via the carbonation of epoxidized soybean oil (ESBO) using carbon dioxide (CO_2_), followed by polymerization with ethylenediamine (EDA) and varying concentrations of 3-aminopropyltriethoxysilane (APTES) (0–20 wt%). The amine groups of EDA and APTES participate in the ring-opening of cyclic carbonates to form β-hydroxyurethane linkages, while the triethoxysilane moieties of APTES may undergo hydrolysis–condensation to produce Si–O–Si domains, resulting in a β-hydroxyurethane–siloxane hybrid network. The optimized formulation CEA-5 exhibited the best, including a tensile strength of 3.3 MPa, elongation at break of ~150%, glass transition temperature (T_g_) of ~7 °C, and thermal stability up to ~350 °C, where major thermal degradation happens. The synthesized coating material also shows adhesion (3.6 MPa on oak for CEA-10), hydrophobic behavior (water contact angle (WCA) ~102° for CEA-5), good chemical and ultraviolet (UV) resistance, and shape memory. The synergistic effect of urethane hydrogen bonding and siloxane crosslinking imparted enhanced toughness, flexibility, and durability. These findings express a scalable, eco-friendly strategy for producing silane-modified NIPU coatings with good mechanical, thermal, and coating performance suitable for sustainable industrial coating and adhesive applications.

## 1. Introduction

Since its discovery by Otto Bayer in 1937, polyurethane (PU) has become one of the most versatile and widely used polymers, valued for its elasticity, toughness, and chemical resistance. It has found broad applications in coatings, foams, sealants, and adhesives [1]. Conventionally, PUs are synthesized through the reaction of diols or polyols (petroleum-based) with diisocyanates, where flexible polyol segments impart elasticity and rigid isocyanate-derived segments contribute mechanical strength, together providing a balance between softness and hardness. However, isocyanates are derived from toxic phosgene gas and pose significant health and environmental hazards [2]. Their reaction with water generates CO_2_ and amines, which can alter polymer morphology and compromise material quality. With increasing emphasis on sustainability, researchers have shifted toward developing greener PU synthesis routes that minimize toxicity while maintaining high performance.

A major approach involves the use of bio-based polyols derived from renewable sources such as vegetable oils, lignin, and vanillin. For example, Patel et al. synthesized a bio-based PU adhesive using castor oil-based polyol and various diols, achieving a maximum mechanical strength of 5.1 MPa, although methylene diphenyl diisocyanate (MDI) was still required as a curing agent [3]. Similarly, vegetable-oil-based PU coatings prepared with hexamethylene diisocyanate (HDI) and MDI were used to investigate the influence of different diols on thermosetting PU properties [4]. To further enhance renewable carbon content, vanillin-based crosslinkers have been incorporated into soybean oil polyol, achieving a tensile strength of 4450 kPa and good thermal stability with a 21% char yield. Despite being bio-derived, these systems still depend on isocyanates and therefore do not fully eliminate toxicity or moisture-sensitivity concerns. Similar trends persist in PU coatings [5]. Wang et al. synthesized a lignin-based PU conjugated with a corrosion inhibitor that served as a bio-based anticorrosive sublayer; however, diisocyanates remained necessary as crosslinking agents [6]. Ha et al. developed a bio-based waterborne PU coating from castor oil and HDI trimer, where isocyanate crosslinking was essential to achieve good transparency, hardness, and antismudge performance through poly(dimethylsiloxane) surface migration [7]. Likewise, Yao et al. formulated a palm kernel oil-based PU coating using MDI as a curing agent for controlled-release fertilizers, achieving a 56-day nutrient release period and enhanced plant growth without toxicity [8]. Further progress has been made through hybrid and functionalized PU systems. Kashyap et al. reported bio-based hybrid PU coatings synthesized from castor oil and isophorone diisocyanate, incorporating molybdenum-modified zeolite as an anticorrosive filler. The optimized formulation (0.5 wt% filler) exhibited corrosion resistance with a corrosion rate of 5.77 × 10^−6^ mm·year^−1^ and a charge transfer resistance of 1.33 × 10^7^ Ω, demonstrating good durability and environmental compatibility [9]. In another study, Li et al. developed a castor-oil-based waterborne polyurethane (WPU) incorporating a vanillin-derived monomer (PHAM) containing reversible imine bonds, enabling heat-triggered self-healing and recyclability. The optimized WPU-PHAM film achieved a tensile strength of 25.9 MPa and >250% elongation, while graphene oxide reinforcement improved both corrosion resistance and mechanical stability [10]. Similarly, Chen et al. synthesized a biomass PU acrylate from jatropha oil and prepared composite coatings by doping monolayer graphene nanoparticles. The optimized coating material achieved a tensile strength of 15.8 MPa, corrosion resistance, and long-term stability under harsh acidic, basic, and saline conditions. Additionally, fluorescent coatings were developed for dual anti-counterfeiting applications, combining sustainability with multifunctional performance [11].

These recent studies highlight substantial progress in bio-based PU research. However, despite using renewable feedstock, most systems still rely on isocyanates as essential precursors, which undermines their environmental and safety advantages. This limitation underscores the need for NIPU systems that eliminate toxic reagents while offering comparable or superior environmental stability and non-moisture sensitivity [1,12,13,14].

To address these limitations, current research has increasingly focused on NIPUs, which rely on the nucleophilic ring-opening addition of amines to five-membered cyclic carbonates, forming β-hydroxyurethane linkages without generating CO_2_ [15,16]. This pathway avoids phosgene-derived diisocyanates and enables tighter control over crosslink density, hydrogen bonding, and network architecture. A wide range of renewable cyclic-carbonate precursors has been developed, including epoxidized-oil carbonates, lignin- and glucose-derived carbonates, and furanic carbonate monomers produced through biomass upgrading [17]. These feedstocks allow systematic tuning of segmental mobility, aromaticity, and hydrophobicity within NIPU matrices. Beyond monomer selection, recent studies have advanced NIPU performance through chemical and physical modifications such as silane-assisted crosslinking, carbonate-functional hyperbranched oligomers, reversible imine or disulfide bonds for dynamic behavior, and nano-reinforcements that enhance barrier properties [18,19,20]. As a result, contemporary NIPU coatings exhibit improved modulus, thermal stability, chemical resistance, and surface energy control compared with earlier bio-PU systems that still rely on diisocyanate-driven hard segment formation. These developments highlight the suitability of NIPUs for high-performance surface applications, particularly where long-term stability, controlled crosslinking, and isocyanate-free processing are critical.

To design effective NIPU materials, various bio-derived substrates have been explored to improve both performance and sustainability. The three main categories of renewable precursors include vegetable oils, lignocellulosic materials, and polysaccharides, each providing functional groups suitable for NIPU formation. For example, bio-based polyester polyols synthesized from glucose [21] and furan-based compounds obtained from lignocellulosic biomass [22] have shown promise as renewable building blocks. Similarly, lignin, cardanol, and limonene, a terpene-derived compound, introduce rigidity, flexibility, and hydrophobicity into NIPU matrices. Moreover, hydroxymethylfuran bis(cyclic carbonate) and 2,5-bis(aminomethyl)furan, both derived from carbohydrate-based biomass, serve as key monomers that enable sustainable NIPU synthesis through eco-friendly, isocyanate-free routes. Among these, vegetable oils stand out as the most abundant and adaptable renewable feedstocks, owing to their inherent reactivity, low cost, and ability to generate cyclic carbonates suitable for high-performance NIPU coatings [23].

In this work, CSBO was synthesized from ESBO using CO_2_ and tetrabutylammonium bromide (TBAB) as a catalyst. The obtained CSBO was then reacted with EDA and varying ratios of APTES to prepare bio-based NIPU coatings. The films were initially cured at room temperature and subsequently thermally cured up to 80 °C. The influence of APTES concentration on the mechanical, thermal, and surface properties was systematically investigated. The developed coatings exhibited tunable flexibility, improved adhesion, and enhanced hydrophobicity, demonstrating their potential as sustainable, high-performance alternatives for advanced coating applications.

## 2. Experimental Details

### 2.1. Materials

ESBO was obtained from Crompton Corporation (Hahnville, LA, USA). TBAB (99%) was purchased from Fisher Scientific (Allentown, PA, USA). EDA was purchased from Sigma-Aldrich (St. Louis, MO, USA). APTES, water, toluene, acetone, *N*,*N*-dimethylformamide (DMF), tetrahydrofuran (THF), dimethyl sulfoxide (DMSO), ethanol, and *N*-methyl-2-pyrrolidone (NMP) were sourced from Fisher Scientific (Allentown, PA, USA). Graphs were generated using Origin 2018 (64-bit, OriginLab Corporation, Northampton, MA, USA).

### 2.2. Synthesis of Carbonated Soybean Oil

**Figure 1:**
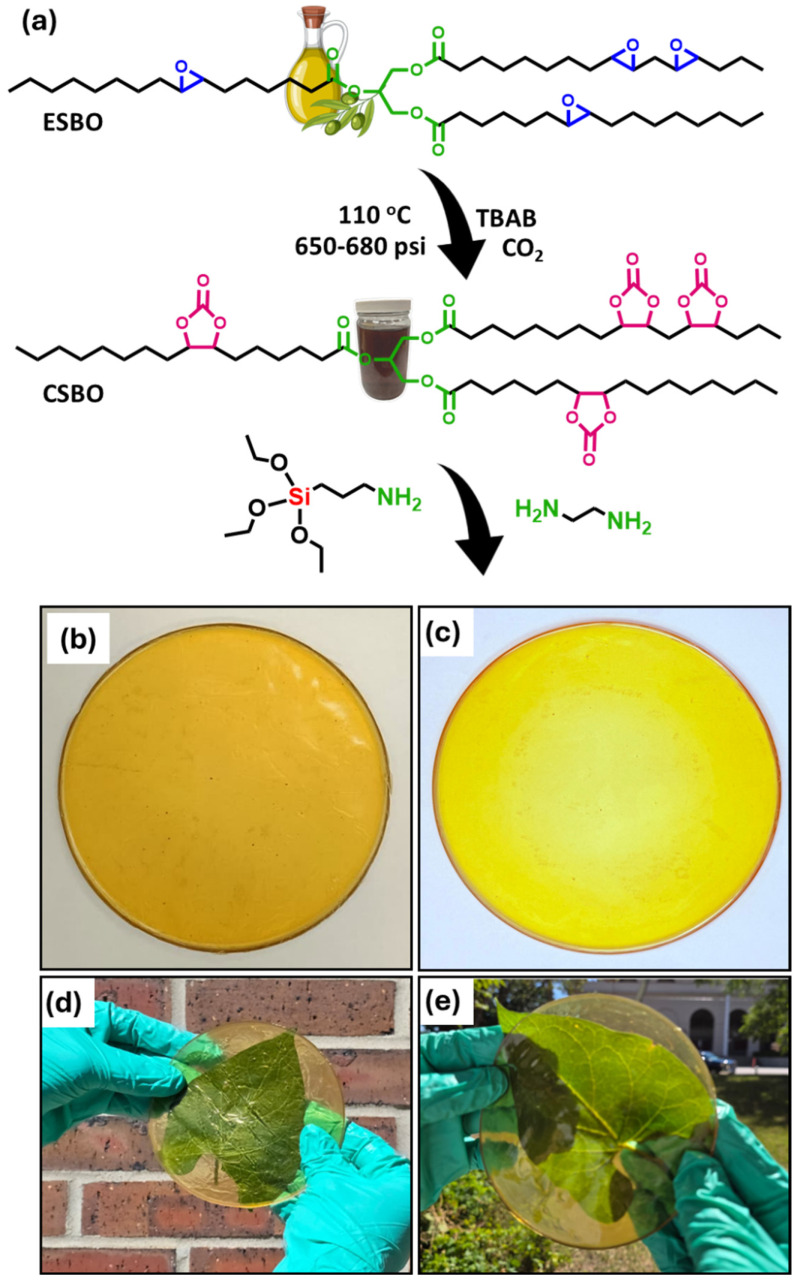
(**a**) Synthesis of CSBO and pictorial representation of the NIPU films. (**b**) CT. (**c**–**e**) CEA-5.

**Figure 2:**
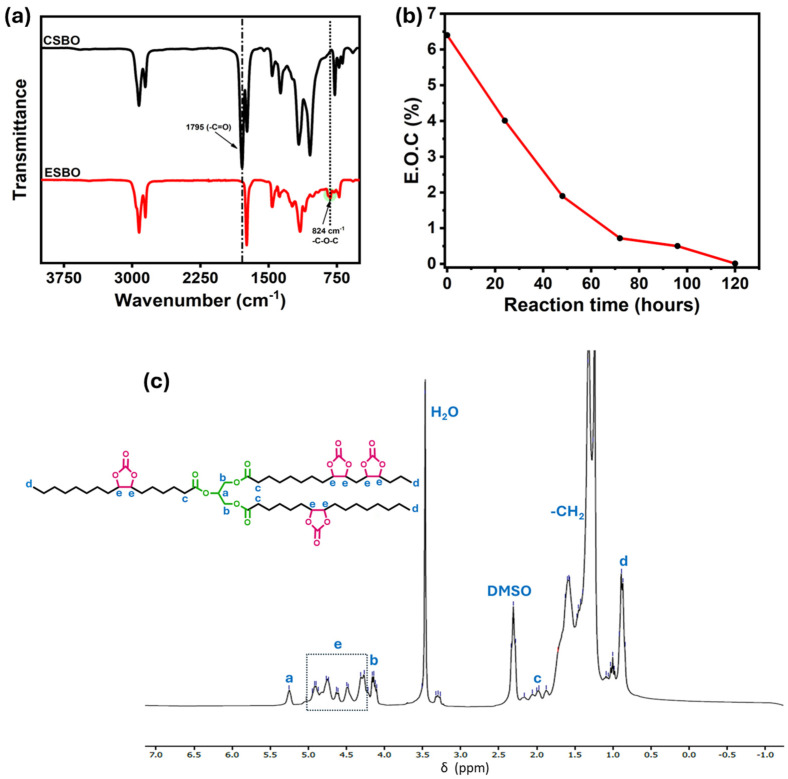
(**a**) FTIR of ESBO and CSBO. (**b**) Epoxy conversion over time. (**c**) ^1^H NMR spectra of CSBO.

Industrial-grade ESBO (epoxy value = 6.4) was employed as the starting material for the synthesis of CSBO. The overall reaction scheme is given in Figure 1a. The carbonation reaction was carried out in a 500 mL Parr high-pressure autoclave reactor. A mixture of ESBO (300 g) and TBAB (11.21 g, 0.011 mol, 0.27 equiv.) was charged into the reactor as the substrate and catalyst, respectively. The system was stirred at 1100 rpm and purged three times with low-pressure CO_2_ to create a uniform and oxygen-free environment. The reactor was then gradually heated to 110 °C. The internal pressure, initially adjusted to 300 psi, increased to 650–680 psi as equilibrium was reached. The progress of the carbonation reaction was monitored by measuring the oxirane oxygen content (EOC%) using Equation (S1). The progressive decline in epoxy concentration over time indicated nearly complete conversion of oxirane groups after approximately 120 h (Figure 2b). Following completion, the system was cooled to 20 °C, and the internal pressure was released gradually. The product obtained was a light-brown, viscous, waxy liquid, confirming the successful carbonation of ESBO to CSBO.

### 2.3. Synthesis of Non-Isocyanate Polyurethane Coating Materials

The synthesis of NIPU films was carried out using CSBO, EDA, and APTES as key components. The ratios of CSBO and EDA were maintained as per previously reported work [24], while APTES was varied to study its effect on film properties. Specifically, 20 wt% of EDA and 5, 10, 15, and 20 wt% of APTES were added relative to CSBO, as summarized in Table S1. For film preparation, 6.0 g of CSBO was placed in a 50 mL beaker, followed by the addition of 1.2 g of EDA (20 wt% of CSBO). Subsequently, different proportions of APTES (0.3, 0.6, 0.9, and 1.2 g corresponding to 5, 10, 15, and 20 wt%) were added to obtain four separate formulations (abbreviated as CEA-5, CEA-10, CEA-15, and CEA-20), along with a control sample abbreviated as CT with 20 wt% EDA without APTES. The mixture was stirred manually using a glass rod until a homogeneous viscous liquid was obtained. To remove trapped air bubbles, the mixture was briefly treated with a heat gun. A Teflon mold was pretreated with a silicon oil as a mold release agent and preheated at 50 °C. The degassed viscous mixtures were then poured into the mold and cured at room temperature for 2 days. This was followed by a stepwise thermal curing process in an oven from 30 °C to 80 °C for 5 days to ensure complete network formation. The resulting films were smooth, transparent, and flexible, confirming the successful synthesis of NIPU coatings. Figure 1b–e represents the pictorial representation of CT and CEA-5 films.

### 2.4. Mechanism of the Synthesis of Non-Isocyanate Polyurethane

The formation of the NIPU network in this system proceeds through two coordinated reaction pathways: nucleophilic ring-opening of the cyclic carbonate groups in CSBO by primary amines, and moisture-triggered hydrolysis and condensation of the triethoxysilane groups of APTES. In the ring-opening reaction, the nitrogen atom of the primary amine in EDA or APTES attacks the electrophilic carbonyl carbon of the cyclic carbonate ring, causing ring cleavage and generating β-hydroxyurethane linkages. Each β-hydroxyurethane unit introduces a hydroxyl group adjacent to the urethane bond, increasing hydrogen-bonding interactions and strengthening cohesion within the polymer matrix. Because EDA contains two amine functionalities, it can react with two cyclic carbonates, effectively bridging multiple CSBO chains and forming a crosslinked β-hydroxyurethane network that establishes the organic backbone of the coating.

APTES participates in the same ring-opening reaction as EDA, forming covalently bound β-hydroxyurethane segments while simultaneously introducing triethoxysilane groups into the polymer network. Although the formulation is not water-based, these triethoxysilane groups readily undergo hydrolysis in the presence of trace ambient moisture, a well-documented behavior of alkoxysilanes. This is supported by Peña-Alonso et al. [25], who demonstrated that APTES converts to silanol species (Si–OH) even under low-moisture conditions. Similar moisture-activated hydrolysis behavior is described by Livage et al. [26] and Tao et al. [27]. In this process, Si–OEt groups convert to silanols with the release of ethanol. During curing, these silanol groups undergo condensation to form Si–O–Si linkages, producing linear or cyclic siloxane structures and partially interconnected inorganic clusters dispersed throughout the β-hydroxyurethane matrix. The characteristic vibrational modes of siloxane networks fall within the 1000–1130 cm^−1^ region, but in hybrid urethane–silane systems they commonly overlap with strong C–O and C–N absorptions, as reported by Innocenzi [28], resulting in a broadened Fourier transform infrared spectra (FTIR) band rather than a distinct Si–O–Si peak.

The siloxane domains generated through condensation become chemically integrated into the β-hydroxyurethane matrix, forming a cohesive organic–inorganic hybrid network. The Si–O–Si linkages improve thermal stability and increase surface hydrophobicity, while residual Si–OH groups can interact with polar substrates and enhance adhesion. The flexible nature of siloxane chains also imparts additional molecular mobility, allowing controlled flexibility within the hybrid structure. Overall, this mechanism provides a sustainable, isocyanate-free route, in which cyclic carbonates serve as benign urethane precursors and APTES contributes both reactive amine functionality and an inorganic siloxane phase. The resulting dual-phase architecture, comprising urethane-rich organic segments and siloxane-rich inorganic domains, supports the observed coating performance and may also contribute to shape-memory behavior through reversible hydrogen-bonding interactions and the mobility associated with flexible Si–O–Si units [25,29,30,31]. The visual representation of the final NIPU material is provided in Supporting Information Figure S1.

### 2.5. Characterizations

The synthetic ESBO and its derivative CSBO were characterized following the standardized procedures established by the American Society for Testing and Materials (ASTM) and the International Organization for Standardization (ISO). The EOC% of ESBO was determined via titration using glacial acetic acid and tetraethylammonium bromide. Rheological properties were analyzed with a TA Instruments AR 2000 ex rheometer (Water Cooperations, New Castle, DE, USA) equipped with a 2° cone plate having a 12.5 mm radius. FTIR spectra were recorded using a PerkinElmer Spectrum spectrometer (PerkinElmer, Inc., Shelton, CT, USA) over the range of 4000–500 cm^−1^ to identify characteristic functional groups in the PU matrix. Proton nuclear magnetic resonance (^1^H NMR) spectra were obtained at room temperature using a Bruker AC-80 spectrometer operating at 400 MHz (Bruker Scientific LLC, Billerica, MA, USA). Deuterated chloroform (CDCl_3_) was used as the solvent, and tetramethylsilane served as the internal reference standard. Sample solutions were prepared at concentrations of 8–10 mg/mL in CDCl_3_. The tensile strength of the NIPU coating materials was evaluated using an Instron 3367 universal testing machine (Instron Corporation, Norwood, MA, USA) operated at a crosshead speed of 50 mm/min. Standard dog-bone-shaped specimens were cut from the thermally cured films, with dimensions of 35 × 4 × ~1 mm (gauge length × width × thickness), following the ASTM D882 standard [32]. Hardness measurements were conducted using a Type D Durometer (PTC Instruments, Los Angeles, CA, USA) in accordance with ASTM D2240 [33]. Thermogravimetric analysis (TGA) was performed on a Discovery Series TGA 550 (TA Instruments, New Castle, DE, USA) to study thermal degradation behavior. Approximately 8–10 mg of sample was heated from 25 °C to 600 °C at a rate of 10 °C/min under a nitrogen flow of 40 mL/min using a platinum pan, and the resulting weight loss versus temperature curve was used for thermal stability assessment. Differential scanning calorimetry (DSC) was carried out on a TA Instruments Q100 DSC (Water Cooperations, New Castle, DE, USA) to determine the T_g_ of PU films. Each 6–8 mg sample was sealed in an aluminum pan and subjected to heating and cooling cycles between −50 °C and 200 °C at a rate of 10 °C/min under a nitrogen atmosphere. WCA measurements were obtained using an Ossila Contact Angle Goniometer (Ossila Ltd., Sheffield, South Yorkshire, UK) to evaluate the surface hydrophobicity of the coatings. The UV exposure test is carried out using a UV lamp of 20 W power at a wavelength of 365 nm.

## 3. Characterization Results and Discussion

### 3.1. Characterizations of the Synthesis of Carbonated Soybean Oil

FTIR plays a crucial role in the structural characterization of ESBO and CSBO (Figure 2a). FTIR enables the identification of key functional group transformations during the carbonation process by monitoring specific vibrational bands. The disappearance of the epoxy ring absorption band near 824 cm^−1^ confirms the consumption of oxirane groups, while the emergence of strong carbonyl stretching vibrations around 1795 cm^−1^ indicates the formation of cyclic carbonate functionalities [34]. Thus, FTIR provides direct molecular evidence of the successful conversion of ESBO to CSBO, rheological (viscosity), and NMR analyses to confirm both chemical modification and structural integrity of the synthesized product.

Viscosity is measured using a TA Instruments AR 2000 ex rheometer, and it increases from 3.7 Pa·s for ESBO to 92.38 Pa·s for CSBO, indicating the formation of a more complex molecular structure [24,35]. This significant rise in viscosity can be attributed to the incorporation of highly polar cyclic carbonate groups, which can promote intermolecular interactions, such as hydrogen bonding, enhance molecular rigidity, and promote chain entanglement. Therefore, the change in viscosity serves as an indirect but reliable indicator of the carbonation reaction’s progression and the successful synthesis of CSBO.

^1^H NMR spectroscopy is essential for structural confirmation as it reveals the chemical environment of hydrogen atoms, helping identify functional groups and verify molecular structure. The ^1^H NMR spectra of CSBO are represented in Figure 2c. The spectra exhibit signals between 4.2 and 5.0 ppm, labeled as “e,” corresponding to the CH protons at the α-position of the cyclic carbonate groups [23]. Additionally, the absence of peaks between 2.5 and 3.0 ppm, typically associated with epoxy protons [36,37], confirms the successful synthesis of CSBO.

### 3.2. Structural Characterizations of Non-Isocyanate Polyurethane Coating Materials

**Figure 3:**
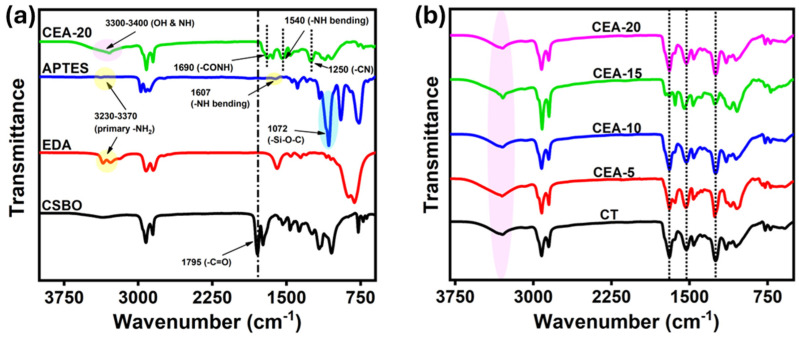
FTIR spectra. (**a**) Comparison of the CEA-20 NIPU materials with monomers. (**b**) Comparison of all the NIPU materials.

The FTIR spectrum of the synthesized NIPU confirms the successful ring-opening reaction (Figure 3a,b). The characteristic cyclic carbonate peak at 1795 cm^−1^ in CSBO [34] disappears in the final NIPU coating after reaction with the primary amines of EDA and APTES. Similarly, the broad band between 3230 and 3370 cm^−1^, attributed to the N-H stretching of primary amines of EDA and APTES, also vanishes, confirming the complete conversion of carbonate groups during the reaction [24]. A new broad band at ~3300–3400 cm^−1^ corresponds to overlapping O-H and N-H stretching vibrations from the newly formed β-hydroxyurethane linkages. The appearance of a strong -CONH stretching band at 1690 cm^−1^ confirms urethane bond formation, while N-H bending vibrations appear at 1540 cm^−1^. Additional peaks at 1250 cm^−1^ are attributed to C-N stretching, confirming the successful synthesis of the NIPU network [36,38].

### 3.3. Mechanical Property of Non-Isocyanate Polyurethane Coating Materials

**Figure 4:**
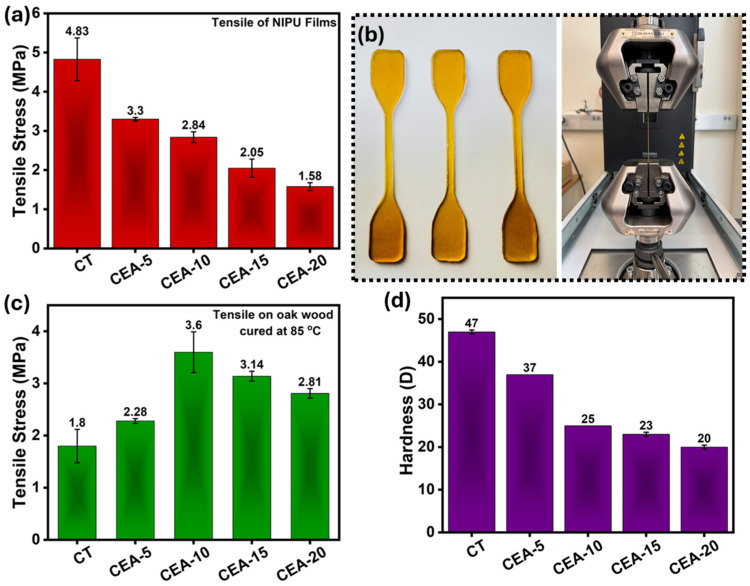
(**a**) Tensile strength of dog-boned-shaped NIPU material. (**b**) Pictorial representation of tensile strength test performance. (**c**) Tensile strength of oak wood adhesive samples. (**d**) Hardness of NIPU materials.

The average tensile strength of all synthesized NIPU films (dog-bone shaped) was measured to evaluate their mechanical properties (Figure 4a,b). The CT sample exhibited the highest tensile strength of 4.83 MPa, consistent with a densely crosslinked β-hydroxyurethane matrix formed solely through the reaction of CSBO with EDA. The high cohesive energy within this organic network arises from extensive hydrogen bonding between urethane and hydroxyl groups, yielding a rigid, compact structure with limited chain mobility [39,40]. Introducing 5 wt% APTES (CEA-5) reduced the tensile strength to 3.3 MPa. This inverse relationship between strength and flexibility reflects a decrease in rigid urethane domain density due to the chain length present in APTES and may be due to Si–O–Si chain formation resulting from the hydrolysis of APTES in the presence of trace amounts of moisture [31,41]. Further increases in APTES content to 10 wt% and 15 wt% (CEA-10 and CEA-15) led to gradual reductions in tensile strength to 2.84 MPa and 2.05 MPa, respectively. These results indicate that the network becomes increasingly dominated by flexible siloxane chains [42,43]. Although the films stay intact and do not break suddenly, the higher amount of soft siloxane domains reduces their ability to carry a load. At higher APTES loadings, the polymer shifts toward an organic–inorganic network with more siloxane content, causing stress to be carried mainly through the siloxane backbone instead of the urethane hard segments. At the highest APTES level (CEA-20), the tensile strength decreased to 1.58 MPa, likely due to excessive siloxane condensation that creates inorganic clusters. The EDA crosslinked system provides rigidity and high cohesive strength, while APTES-modified hybrids introduce elasticity and toughness through siloxane flexibility [44]. Optimal mechanical performance, balancing moderate tensile strength (~3 MPa) with elongation (~150%), is achieved at 5 wt% APTES (CEA-5), where homogeneous dispersion of siloxane crosslinks occurs within the β-hydroxyurethane matrix. These findings demonstrate that controlled APTES incorporation effectively tunes the mechanical behavior of bio-based NIPU coatings.

The elongation characteristics of the CSBO-based NIPU films, synthesized from EDA and varying proportions of APTES, are illustrated in Figure S2. The stress–strain profiles reveal a clear and systematic evolution of flexibility and ductility with increasing APTES concentration. The CT sample displayed a relatively low elongation at break of ~60%, indicative of a stiff and brittle polymeric network. This behavior arises from the dense β-hydroxyurethane crosslinks formed between CSBO and EDA. The bifunctional EDA bridges multiple carbonate groups, yielding a highly hydrogen-bonded, tightly packed matrix that restricts chain mobility and limits plastic deformation before fracture. Incorporation of 5 wt% APTES (CEA-5) produced an improvement in elongation, increasing the strain at break to approximately 150% more than double that of the control. This increased flexibility is due to the longer chain in APTES and may also result from the formation of Si–O–Si siloxane linkages during curing [45]. The flexible siloxane backbone acts as a soft segment within the β-hydroxyurethane framework, relieving internal stress and allowing reversible segmental motion under tension. As the silane content increased to 10 wt% and 15 wt% (CEA-10 and CEA-15), the elongation remained high above 100% in both cases, though slightly lower than for CEA-5. This behavior suggests that a balanced distribution of organic (β-hydroxyurethane) and inorganic (siloxane) domains supports both extensibility and structural integrity. At 20 wt% APTES (CEA-20), the elongation dropped to about 90%, indicating reduced flexibility. This decrease is likely due to inorganic-rich regions that hinder uniform stretching and create stress-concentration points that limit elongation. In summary, the elongation results clearly demonstrate that APTES incorporation transforms the brittle urethane matrix into a tunable, flexible hybrid polymer. Moderate APTES loading (5–10 wt% CEA-5) achieves a balance between crosslink density and molecular mobility.

The tensile shear strength of the synthesized NIPU material was evaluated on oak wood substrates to assess adhesion, with samples cured at 85 °C (Figure 4c). For the test, a 27.5 mm × 27.5 mm (length × breadth) area was covered with adhesives and cured at 85 °C. To determine the average adhesive tensile strength, all samples were prepared in the same manner, and three specimens were prepared for each sample. The adhesive strength varied systematically with APTES concentration, reflecting the influence of the hybrid β-hydroxyurethane–siloxane network on both interfacial adhesion and cohesive integrity [23]. The CT exhibited an adhesive strength of 1.8 MPa through the reaction between the cyclic carbonate groups of CSBO and the primary amines of EDA. Adhesion in this sample was achieved through hydrogen bonding and polar interactions between the urethane and hydroxyl groups in the polymer and the surface hydroxyls on the oak substrate. Incorporating 5 wt% APTES (CEA-5) increased the adhesive strength to 2.28 MPa, showing better bonding with the wood surface. The amine group in APTES reacts with the cyclic carbonates to form β-hydroxyurethane linkages, while the triethoxysilane groups can hydrolyze and condense to form Si–O–Si and Si–O–C bonds. These covalent bonds act like molecular bridges between the polymer and the wood, which improves the overall adhesion strength. The highest adhesion strength of 3.6 MPa was observed for CEA-10, confirming that this composition provides an optimal balance between network cohesion and interfacial bonding. The combined effect of organic urethane hydrogen bonding and inorganic siloxane coupling leads to strong and durable adhesive joints. When the APTES concentration was further increased to 15 wt% and 20 wt% (CEA-15 and CEA-20), the adhesive strength decreased slightly to 3.14 MPa and 2.81 MPa, respectively. This reduction is attributed to the brittle behavior of NIPU material. These rigid clusters reduce flexibility and hinder uniform stress distribution along the adhesive–substrate interface, causing premature debonding under tensile load. This observation agrees with previous reports on hybrid urethane–silane adhesives, where silane modification improves bonding to polar substrates such as wood, glass, and metal through enhanced chemical compatibility and interfacial energy matching [46]. Table 1 shows the comparison of the work of PU and NIPU materials in some recent studies.

The Shore D hardness results for the CSBO-based NIPU films are presented in Figure 4d. The measurements reveal a progressive decrease in surface hardness with increasing APTES concentration, reflecting the structural transition from a rigid β-hydroxyurethane network to a flexible organic–inorganic hybrid system. The CT sample exhibited the highest hardness of 47.3, confirming the formation of a densely crosslinked and hydrogen-bonded β-hydroxyurethane matrix. The reaction between CSBO and EDA generates short, stiff polymer chains containing abundant urethane and hydroxyl groups that engage in extensive intermolecular hydrogen bonding. This compact network restricts chain motion and enhances segmental rigidity, producing a hard, glassy surface. Introducing 5 wt% APTES (CEA-5) decreased the hardness to 37 D, indicating disruption of rigid urethane domains. The formation of Si–O–Si linkages through hydrolysis and condensation of APTES introduces flexible siloxane segments that act as soft crosslinks within the β-hydroxyurethane framework. These segments lower the cohesive-energy density of the matrix and permit limited molecular mobility, yielding a softer yet coherent coating. Further increases in APTES to 10 wt% and 15 wt% (CEA-10 and CEA-15) produced a more pronounced reduction in hardness to 25 and 23, respectively. At the highest silane loading (CEA-20), the hardness declined further to 20.7 D, attributed to excessive siloxane condensation and partial phase separation that makes this material more flexible. Overall, the hardness results correlate well with the tensile and elongation data, confirming a strength–flexibility by the interplay between β-hydroxyurethane hydrogen bonding and siloxane flexibility [23].

### 3.4. Thermal Property of Non-Isocyanate Polyurethane Coating Materials

**Figure 5:**
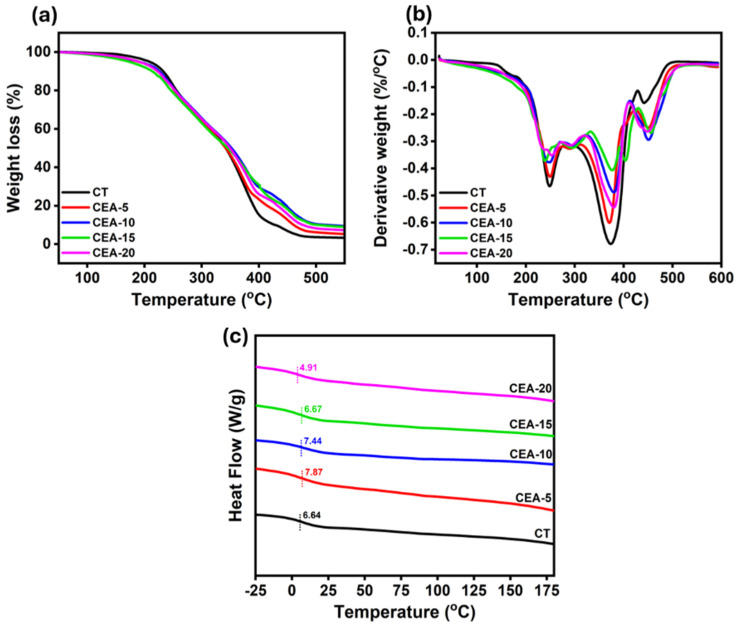
(**a**) TGA, (**b**) DTG, and (**c**) DSC analysis of NIPU materials.

The TGA and derivative thermogram (DTG) curves of the CSBO-based NIPU coatings (Figure 5a,b) reveal the influence of APTES incorporation on the thermal stability of the system. All formulations CT and APTES-modified hybrids NIPU materials 5, 10, 15, and 20 wt% (CEA-5, CEA-10, CEA-15, CEA-20) exhibited a principal degradation stage between 250 and 350 °C, attributed to the breakage of urethane linkage, leaving less than 10% residue at 450–500 °C, attributed to the decomposition of char residues. Another degradation between 350 and 450 °C could be due to the soft aliphatic segments of the polymer backbone, such as carbon chain length from CSBO [24,57,58]. The CT film showed the earliest mass-loss onset and the most intense DTG peak near 350–450 °C. Incorporation of 5 wt% APTES (CEA-5) slightly delayed the onset of degradation and broadened the DTG peak, indicating improved thermal stability. The flexible Si–O–Si linkages formed by APTES hydrolysis and condensation acted as thermally stable domains that dissipated localized stress and delayed chain scission. At intermediate APTES loadings (10–15 wt%), the TGA/DTG profiles displayed further delayed T_5%_ values and broader decomposition ranges, confirming the formation of an organic–inorganic network. The siloxane domains absorbed heat and restricted segmental motion, while β-hydroxyurethane segments maintained structural cohesion. Such behavior agrees with prior reports showing that hybrid siloxane linkages enhance degradation onset while reducing modulus through flexible segment incorporation [59]. At the highest APTES concentration (CEA-20), thermal improvement became marginal: the degradation onset was similar to that of the control, though the residual mass was slightly higher. This behavior suggests that excess siloxane condensation and phase separation hinder uniform heat transfer, forming domains prone to localized decomposition. The mechanical softness (tensile strength 1.58 MPa, hardness 20 D) and moderate elongation (~90%) further support this structural heterogeneity. These findings agree with previous studies [60,61], which reported that hydrogen bonding in β-hydroxyurethane linkages stabilizes NIPUs up to ~300 °C despite the weakening effect of adjacent hydroxyl groups on bond dissociation energy. In the present system, incorporation of siloxane domains enhances heat-transfer resistance.

The DSC thermograms of the CSBO-based NIPU coatings (Figure 5c) exhibit single, broad glass-transition regions for all formulations, confirming the formation of homogeneous polymer networks without visible phase separation between organic and inorganic domains. The measured T_g_ were 6.64 °C (CT), 7.87 °C (CEA-5), 7.44 °C (CEA-10), 6.67 °C (CEA-15), and 4.91 °C (CEA-20). The CT sample displayed a T_g_ of 6.64 °C, characteristic of a crosslinked β-hydroxyurethane network formed through the reaction of CSBO cyclic carbonates with EDA amines. The low T_g_ reflects the flexible aliphatic segments of the soybean–oil backbone, while the distinct transition indicates a uniform structure stabilized by hydrogen bonding between urethane and hydroxyl groups. Incorporation of 5 wt% APTES (CEA-5) increased T_g_ to 7.87 °C, suggesting restriction in chain mobility due to Si–O–Si siloxane crosslink formation during APTES hydrolysis and condensation. These inorganic linkages strengthen the polymer network, which explains the higher elongation (about 150%) and moderate tensile strength (around 3.3 MPa). The Tg elevation indicates that siloxane domains are well dispersed within the urethane matrix, forming a compact hybrid structure that resists thermal motion without inducing phase separation. At 10 wt% APTES (CEA-10), T_g_ remained nearly unchanged (7.44 °C), implying a balance between stiff β-hydroxyurethane segments and flexible siloxane chains. The consistent T_g_ suggests that organic and inorganic components form a hybrid network, maintaining uniform segmental dynamics and cohesive interactions throughout the polymer. Further increasing APTES content to 15 wt% and 20 wt% (CEA-15, CEA-20) led to gradual T_g_ decreases to 6.67 °C and 4.91 °C, respectively. This reduction is due to flexible siloxane chains and phase separation caused by excessive APTES condensation. The resulting siloxane-rich regions introduce localized free volume, increase molecular mobility, and consequently lower T_g_. The broadened transitions also indicate heterogeneous relaxation behavior, typical of soft hybrid polymer systems. Taken together, the DSC results are consistent with the mechanical and TGA findings. Moderate APTES incorporation (5–10 wt%) produces a homogeneous organic–inorganic hybrid network that maintains cohesive thermal transitions and balanced mechanical performance, while higher silane contents yield softer, more flexible materials at the expense of structural rigidity. Similar T_g_ behavior has been reported for other bio-based NIPUs containing siloxane or polyether segments, where the T_g_ depends primarily on crosslink density and segmental mobility [61,62]. The overall thermal characteristics are represented in Table S2. T_HRI_ is calculated using the following Equation (S2).

### 3.5. Gel Fraction and Degree of Swelling Test

**Figure 6:**
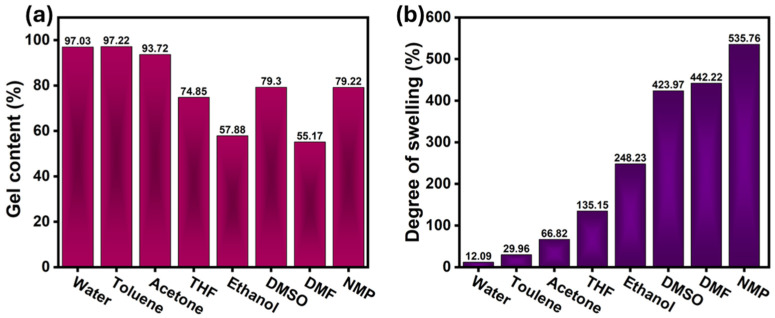
(**a**) Gel fraction and (**b**) degree of swelling test of the CEA-5 NIPU material.

The gel fraction and degree of swelling analyses were conducted to evaluate the crosslink density and solvent resistance of the CSBO-based NIPU network containing 5 wt% APTES (CEA-5), which previously exhibited the best balance between mechanical flexibility and adhesive strength. For the test, the samples were immersed in eight different solvents for 24 h. These solvents were categorized based on their polarity and protic characteristics as follows: polar aprotic solvents (THF, DMF, acetone, NMP, and DMSO), polar protic solvents (water and ethanol), and a nonpolar solvent (toluene). After immersion, the samples were wiped with tissue, and their weight was recorded. The samples were then oven-dried at 75 °C for 48 h to remove any residual solvent. The gel fraction and degree of swelling were then determined using Equations (S3) and (S4), respectively [4]. Figure 6a,b summarize the gel fraction and degree of swelling values, respectively, of the cured film in various solvents with differing polarity and solubility parameters. The gel fraction of the CEA-5 sample ranged from 55.17% (in DMF) to 97.22% (in toluene). High gel fractions in solvents such as toluene (97.22%) and water (97.03%) indicate a densely crosslinked network with resistance to dissolution. This stability arises from the strong β-hydroxyurethane linkages and Si–O–Si siloxane bridges that reinforce the polymer matrix. In contrast, polar aprotic solvents such as DMF (55.17%) and DMSO (79.3%) showed lower gel fraction and higher sol fractions, reflecting partial network relaxation and solvent penetration via hydrogen bonding with urethane and hydroxyl groups. Similar solvent-dependent trends have been reported for hybrid NIPU and PU–siloxane systems, where solvent polarity and hydrogen-bonding capability strongly influence extraction and gel fraction behavior. The degree of swelling followed the order: NMP (535.76%) > DMF (442.22%) > DMSO (423.97%) > Ethanol (248.23%) > THF (135.15%) > Acetone (66.82%) > Toluene (29.96%) > Water (12.09%). This trend correlates with solvent polarity and hydrogen-bonding parameters: highly polar solvents such as NMP and DMF easily penetrate and expand the polymer network by disrupting intermolecular hydrogen bonds. Moderate swelling in ethanol reflects its dual character as a polar protic solvent; its hydrogen bonding promotes polymer–solvent interactions, but its lower dielectric constant compared to NMP or DMSO limits chain separation. Conversely, minimal swelling in water and toluene underscores the semi-hydrophobic nature of the network, imparted by long aliphatic chains from CSBO and siloxane domains, consistent with the coating’s durable and water-repellent behavior. From a structure–property standpoint, the moderate crosslink density inferred from the gel fraction analysis complements the mechanical and thermal trends discussed earlier. The CEA-5 formulation exhibited high elongation (~150%) and intermediate tensile strength (~3.3 MPa), typical of flexible yet cohesive hybrid networks. Siloxane linkages introduced by APTES impart elasticity and permit controlled swelling without structural collapse, while β-hydroxyurethane hydrogen bonding ensures cohesion. The partial solvent uptake in polar media confirms the presence of accessible hydroxyl groups, which likely contribute to the strong interfacial adhesion to wood substrates. Collectively, the gel fraction and swelling results confirm that the CEA-5 hybrid NIPU forms an organic–inorganic network with tunable solvent resistance. The dense siloxane backbone ensures stability in nonpolar environments, while accessible hydroxyl groups enhance reactivity toward polar substrates. Similar dual-character NIPUs have been reported, where optimized silane incorporation yields enhanced flexibility and solvent resistance. In conclusion, the gel fraction and swelling behavior reaffirm that the CEA-5 formulation achieves an ideal balance between crosslink density and chain mobility, underlying its mechanical flexibility, moderate hardness, and adhesive performance. The strong consistency across mechanical, thermal, and solvent-resistance data demonstrates the effectiveness of APTES in generating a chemically stable, bio-based NIPU network suitable for durable coating and wood-adhesive applications under diverse environmental conditions. For comparison, the gel fraction and degree of swelling of CT, CEA-10, CEA-15, and CEA-20 samples are given in supporting information Figure S3. The FTIR spectra of all samples recorded after the gel fraction and swelling tests showed no noticeable changes, indicating the chemical stability of the polymer network. This suggests that solvent exposure did not alter the structural integrity or functional groups of the NIPU coating (Figure S4a,b).

The UV exposure test is conducted to evaluate the photostability and weathering resistance of the coating material, as prolonged UV radiation can induce polymer degradation through chain scission or oxidation [63]. After 40 days of UV exposure, no significant changes were observed in the FTIR spectra of the samples, confirming the UV stability and chemical durability of the NIPU coating structure (Figure S4c). In addition, TGA, DTGA, and DSC analyses were performed, and no noticeable changes were observed compared with the fresh samples. The thermal profiles remained almost identical, indicating that the material is stable under UV exposure (Figure S5).

### 3.6. Contact Angle Measurement

The surface wettability of the CEA-5 NIPU coating material was evaluated using static WCA measurements to assess its hydrophobic–hydrophilic balance [64]. The average WCA recorded for the CEA-5 film was 102.34° (Figure S6b), indicating a hydrophobic surface. This degree of hydrophobicity arises from the combined effects of the long aliphatic chains of the CSBO backbone and the siloxane groups introduced through APTES modification. The hydrocarbon chains derived from CSBO lower the surface energy, while the Si–O–Si siloxane structures generated during APTES condensation further enhance water repellency. During film formation and curing, siloxane domains tend to migrate toward the air–film interface, enriching the surface with nonpolar groups and thereby increasing the contact angle. The observed WCA exceeding 100° demonstrates that the surface effectively resists wetting, an essential attribute for protective coating and adhesive applications. The siloxane-enriched surface layer acts as a barrier against moisture uptake, whereas the underlying β-hydroxyurethane matrix maintains cohesive integrity through hydrogen bonding and moderate crosslinking. This surface–bulk synergy corresponds closely with the mechanical, adhesive, and swelling behavior discussed previously, where the same CEA-5 formulation exhibited optimal flexibility, moderate hardness, and strong interfacial adhesion. The hydrophobic outer layer complements these properties by providing environmental durability and resistance to water penetration without compromising adhesion to substrates such as wood. Such hydrophobic behavior is typical of hybrid silane-modified NIPUs, which generally exhibit WCAs between 95° and 110° due to surface enrichment by siloxane and aliphatic moieties. In conclusion, the contact-angle analysis confirms that the CEA-5 NIPU coating possesses a hydrophobic yet structurally stable surface, making it a promising bio-based, moisture-resistant material for protective and adhesive coating applications. Moreover, the other samples were also tested for WCA, as shown in Figure S6.

### 3.7. Ink Contraction Behavior/Color Repellency Test

**Figure 7:**
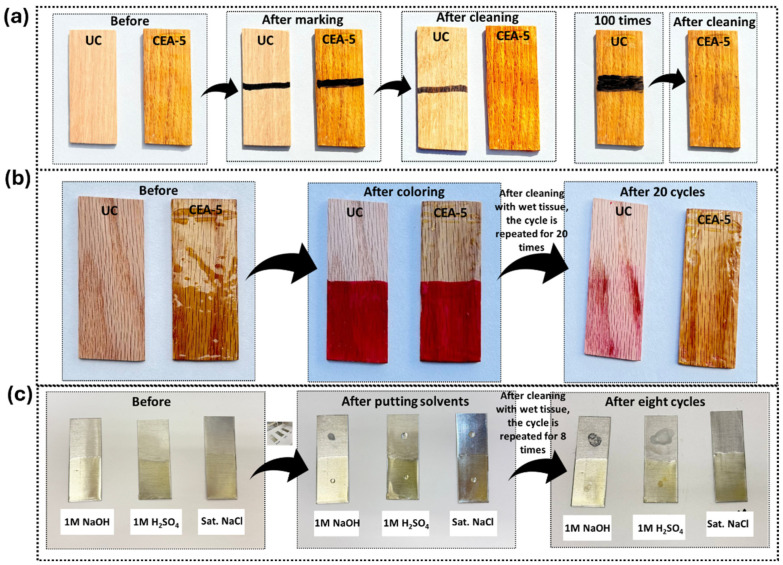
(**a**) Ink contraction behavior, (**b**) color repellency test, and (**c**) chemical resistance test of the CEA-5 NIPU material.

Ink and stain resistance testing was conducted as a qualitative evaluation of the surface integrity, impermeability, and chemical repellency of the NIPU coatings on wood substrates. Two types of samples were examined: uncoated wood (abbreviated as UC in Figure 7) and wood coated with the CEA-5 NIPU formulation. In the first test, an oil-based marker was applied to both surfaces and subsequently wiped with a wet tissue. The uncoated wood absorbed the ink immediately, leading to permanent staining due to its high porosity and surface energy. In contrast, the NIPU-coated surface exhibited ink repellency. The ink marks were completely removed upon wiping, leaving no visible trace even after repeated cycles (Figure 7a). In the second test, a red water-based dye was applied to both uncoated and coated surfaces to evaluate resistance against aqueous staining. Upon wiping, the uncoated wood retained a noticeable red stain, whereas the coated surface remained clean and unaffected. This process was repeated for 20 cycles, and no visible color retention or surface discoloration was observed on the coated samples throughout the test (Figure 7b). These results demonstrate that the NIPU coating forms a continuous, defect-free, and hydrophobic barrier layer, effectively sealing the wood pores and preventing the infiltration of liquids. This performance reflects film formation, surface smoothness, and chemical resistance attributes essential for protective and decorative coatings such as furniture finishes, flooring, and outdoor wood protection [65].

### 3.8. Chemical Resistance Test

Chemical resistance testing is a critical evaluation used to determine the durability and environmental stability of coating materials under aggressive chemical conditions. NIPU coatings are often intended for applications exposed to corrosive or chemically harsh environments; therefore, assessing their resistance to acids, bases, and salts is essential to ensure long-term performance. In this study, stainless-steel coupons were partially coated with the optimized NIPU formulation (CEA-5) (Figure 7c). The coated samples were exposed to 1 M H_2_SO_4_, 1 M NaOH, and a saturated NaCl solution to represent acidic, alkaline, and neutral environments, respectively, conditions commonly encountered in industrial, marine, and atmospheric settings. A high level of resistance in such environments signifies a robust polymer network with strong chemical bonding and minimal susceptibility to hydrolysis or corrosion, both of which are critical for protective and sustainable coatings. For each test, a drop of the chemical solution (acidic, basic, or neutral) was carefully placed on both coated and uncoated regions of the stainless-steel coupons. After natural drying, the surfaces were gently wiped with a wet tissue. This process was repeated for eight consecutive cycles to assess long-term durability. The coated regions exhibited no visible changes in color, surface morphology, or signs of degradation or corrosion up to seven exposures, demonstrating the chemical resistance of the NIPU coating. In contrast, uncoated surfaces displayed noticeable corrosion spots and discoloration after only one cycle of exposure to acidic and basic solutions, confirming the protective efficiency of the CEA-5 coating. No visible changes were observed on either coated or uncoated regions in the neutral salt medium, indicating overall chemical stability under non-corrosive conditions. The outstanding chemical resistance of the NIPU coating can be attributed to its highly crosslinked polymeric network and the presence of urethane and siloxane linkages, which impart strong chemical stability and interfacial adhesion to the substrate. The absence of degradation in acidic and alkaline media suggests that the dense network effectively restricts the diffusion of corrosive species. The urethane and carbonate functionalities form hydrogen-bonded and covalently crosslinked domains that minimize hydrolytic attack and limit chain mobility, thereby enhancing chemical inertness. Moreover, the silane component likely promotes the formation of a Si–O–metal interfacial layer, further improving barrier performance and adhesion to stainless steel. Overall, the comparison between coated and uncoated regions confirms that the NIPU film acts as an effective protective barrier, preventing acid- or base-induced corrosion even after repeated exposure cycles. The unaltered surface appearance in saline conditions further demonstrates the coating’s stability in neutral environments, highlighting its suitability for marine and industrial coating applications where exposure to chemically diverse media is common [66].

### 3.9. Shape Memory Observation

**Figure 8:**
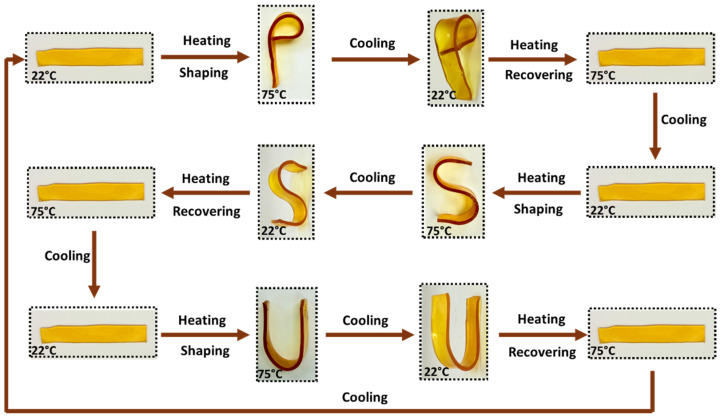
Pictorial representation of the shape memory behavior of the CEA-5 NIPU material.

The NIPU material exhibited shape memory behavior, demonstrating its ability to recover its original shape after deformation when exposed to an external stimulus such as heat [67]. This reversible shape transformation indicates the presence of a dynamic crosslinked network within the polymer matrix, allowing temporary shape fixation and subsequent recovery, which highlights the material’s potential for smart coating and responsive material applications. For the shape memory evaluation, the NIPU specimen was heated above its T_g_ to activate the molecular mobility of the polymer chains. The rectangular-shaped sample was heated at 75 °C for 2 min, reshaped into the letter “P”, and subsequently cooled to room temperature to fix the temporary shape. When reheated to 75 °C for 2 min, the material recovered its original rectangular geometry. This reversible behavior arises from the dual-segment structure of the NIPU network: the urethane linkages provide a stable, permanent crosslinked framework, while the dynamic Si–O–Si bonds formed from the condensation of APTES enable reversible bond exchange and network rearrangement upon heating. As a result, the material exhibits shape memory performance and thermally responsive elasticity, as illustrated in Figure 8.

## 4. Conclusions

This study successfully demonstrates the development of silane-modified bio-based NIPU coatings synthesized from CSBO, EDA, and APTES. The environmentally benign ring-opening reaction between cyclic carbonate and amine groups yielded a β-hydroxyurethane–siloxane hybrid network with tunable properties. Structural analyses confirmed complete conversion of carbonate functionalities and effective integration of Si–O–Si linkages, which imparted flexibility, hydrophobicity, and enhanced adhesion. The optimized CEA-5 formulation exhibited a balance of mechanical and surface performance, achieving ~3.3 MPa tensile strength, ~150% elongation, T_g_ ~7 °C, and thermal stability up to 350 °C. Furthermore, the hybrid coating displayed strong adhesion (3.6 MPa on oak wood), chemical resistance in acidic, basic, and saline environments, a high WCA (~102°), and UV stability. The synergistic interaction between β-hydroxyurethane hydrogen bonding and siloxane crosslinks provided durability, solvent resistance, and reversible shape-memory behavior. These findings confirm that controlled silane incorporation effectively tailors the balance between rigidity and elasticity in NIPU systems. Moreover, future work may explore the functionalization of these NIPU coatings with pigments, conductive fillers, or biocidal additives to expand their potential applications. Overall, this work establishes a sustainable and scalable approach for producing eco-friendly, NIPU coatings with robust thermal, mechanical, and surface performance, suitable for protective, adhesive, and smart coating applications in industrial and environmental settings. Although the coating demonstrated promising mechanical, thermal, and UV stability, including 40 days of continuous UV exposure, long-term durability under broader operational conditions, such as humidity cycling, temperature fluctuations, and extended weathering, will be investigated in future work to fully assess its real-world performance.

## Figures and Tables

**Table 1:** Comparison of the previously reported work on PU and NIPU materials.

Sr. No.	Bio-Based Material	Fillers	Substrate	Tensile Strength (MPa)	Reference DOI
1	Castor oil (50%)	HDI, cellulose Acetate	Poplar wood	2.84	[47]
2	Castor oil (20%)	HDI, cellulose Acetate	Poplar wood	2.37	[47]
3	Castor oil (70%)	HDI, cellulose Acetate	Poplar wood	0.94	[47]
4	Castor oil	Cadaverine + Nvoc-Cl	Polyethylene	4.6	[48]
5	Castor Oil	Cadaverine + Nvoc-Cl	Oak wood	2	[48]
6	Palm oil-based polyester polyol with MDI	Glycerol	Teak wood	5.3	[49]
7	Palm oil-based polyester polyol with toluene diisocyanate	Glycerol	Teak wood	4.7	[49]
8	Palm oil polyol	-	Hard wood	2.6	[50]
9	Jatropha oil polyol	-	Hard wood	4.9	[50]
10	Canola polyol	-	Birch wood	5.7	[51]
11	Castor Oil	-	Wood	2.19	[52]
12	Castor Oil	-	Pinewood	1.9	[53]
13	COP	*N*, *N*-bis(2-hydroxyethyl) thiophene-2,5-dicarboxamide	Oak wood	7.22	[54]
14	COP + ETAM	*N*, *N*-Bis(2-hydroxyethyl)-terephthalamid	Oak wood	9.68	[54]
15	poly(propylene glycol dicyclic carbonate)	APTES	Stainless steel	2.7	[55]
16	CSBO	EDA	Oak wood	6.23	[24]
17	CSBO	Butane diamine	Oak wood	6.23	[24]
18	CSBO	Hexamethylene diamine	Oak wood	6.23	[24]
19	CSBO	Butane diamine	Film	1.1	[56]
20	CSBO	Pentane diamine	Film	1.0	[56]
21	CSBO	Octane diamine	Film	1.2	[56]
22	Carbonated algae oil	Butane diamine	Film	0.5	[56]
23	Carbonated algae oil	Pentane diamine	Film	0.3	[56]
24	CSBO	EDA	Film	1.97	[24]
25	CSBO	Butane diamine	Film	0.59	[24]
26	CSBO	Hexamethylene diamine	Film	0.22	[24]
27	CSBO	EDA	Film	4.83	[This work]
28	CSBO	EDA + APTES	Film	3.3	[This work]
29	CSBO	EDA	Oak wood	1.8	[This work]
30	CSBO	EDA + APTES	Oak wood	3.6	[This work]

## Data Availability

The original contributions presented in this study are included in the article/Supplementary Materials. Further inquiries can be directed to the corresponding authors.

## Supplementary Materials

The following supporting information can be downloaded at https://www.mdpi.com/article/10.3390/ma18245551/s1, Figure S1: Visual representation of the final NIPU material; Figure S2: Elongation of (a) CT (b) CEA-5 (c) CEA-10 (d) CEA-15 (e) CEA-20 NIPU samples; Figure S3: Gel fraction and degree of swelling of (a,b) CT, (c,d) CEA-10, (e,f) CEA-15 and (g,h) CEA-20 samples respectively; Figure S4: (a,b) FTIR spectra after gel fraction and degree of swelling test of CEA-5 NIPU material (c) FTIR spectra after UV exposure test of all NIPU materials; Figure S5: (a) TGA (b) DTGA and (c) DSC spectra of NIPU materials after UV exposure; Figure S6: WCA of (a) CT (b) CEA-5 (c) CEA-10 (d) CEA-15 and (e) CEA-20 NIPU materials; Table S1: Formulation table of the synthesis of NIPU coating materials; Table S2: Thermal characteristics of NIPU materials.
