# Supplementary material for: Design and Characterization of Silane-Modified Bio-Based Non-Isocyanate Polyurethane Coatings for Advanced Surface Applications
Source: Materials (Basel). 2025 Dec 10;18(24):5551. doi: 10.3390/ma18245551 (PMC12734775; doi:10.3390/ma18245551)
Supplement: Supplementary file 1 [file materials-18-05551-s001.zip › materials-3996310-supplementary.pdf]

Design and Characterization of Silane-Modified Bio-Based Non-Isocyanate Polyurethane Coatings for Advanced Surface Applications

Rutu Patel[1*], Ajay Kumar[1,2], Mayankkumar L. Chaudhary[1*], Ram K. Gupta[1,3*]
[1]National Institute for Materials Advancement, Pittsburg State University, 1204 Research Road, Pittsburg, KS 66762, USA
[2]Department of Physics, Pittsburg State University, 1701 S Broadway St, KS, 66762
[3]Department of Chemistry, Pittsburg State University, 1701 S Broadway St, KS, 66762
*Corresponding author: rutupatel114538@gmail.com; mayank.chaudhary1807@gmail.com; ramguptamsu@gmail.com

Equation S1. $EOC(\%) = \frac{V \times N \times 1.6}{Wt.sample}$

where V is the titration volume (mL) and N is the normality of perchloric acid ($HClO_4$).

Equation S2. $T_{HRI} = 0.49[T_{-5\%} + 0.6(T_{-30\%} - T_{-5\%})]$

Equation S3. $Gel\ fraction\ (\%) = \frac{m_2}{m_0} \times 100$

Where m_2 is the weight after drying.

Equation S4. $Degree\ of\ swelling(\%) = \frac{m_1 - m_0}{m_0} \times 100$

Where m_0 the initial is weight and m_1 is the weight after swelling.

Table S1. Formulation table of the synthesis of NIPU coating materials.

Sr. No.	Materials		Sample Name				
			**CT**	**CEA-5**	**CEA-10**	**CEA-15**	**CEA-20**
1	CSBO (gm)		6	6	6	6	6
2	20wt% EDA (gm)		1.2	1.2	1.2	1.2	1.2
3	APTES	gm	-	0.3	0.6	0.9	1.2
		Wt.%	-	5	10	15	20

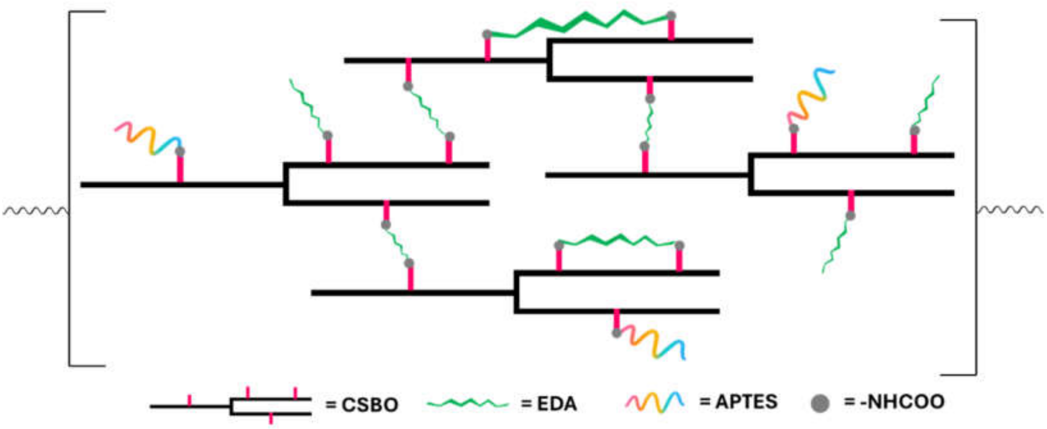

Figure S1. Visual representation of the final NIPU material

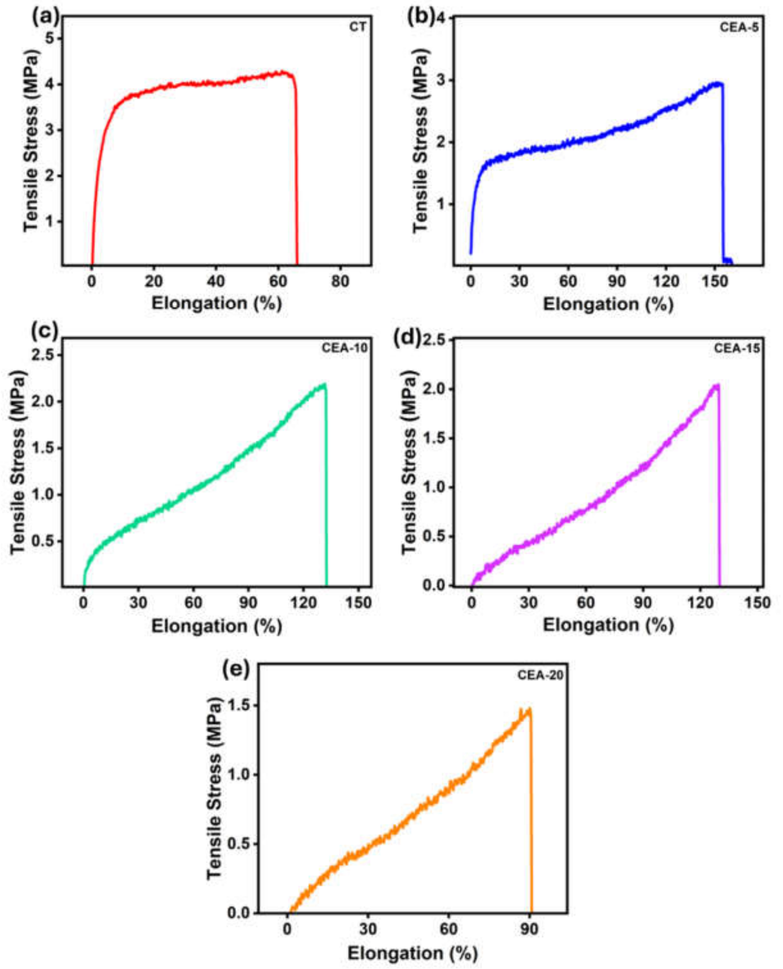

Figure S2. Elongation of (a) CT (b) CEA-5 (c) CEA-10 (d) CEA-15 (e) CEA-20 NIPU samples

Table S2. Thermal characteristics of NIPU materials.

Sr. No.	Sample Name	$T_{5\%}$ (°C)	$T_{30\%}$ (°C)	T_{max} (°C)	T_{HRI}	T_g (°C)	Residual Mass (%)
1.	CT	208.45	285.24	373.52	124.72	6.64	2.88
2.	CEA-5	188.92	281.47	370.72	119.78	7.87	4.25
3.	CEA-10	190.58	286.06	379.79	121.45	7.44	8.81
4.	CEA-15	172.68	278.45	376.85	115.70	6.67	8.13
5.	CEA-20	190.10	284.75	379.79	120.98	4.91	6.49

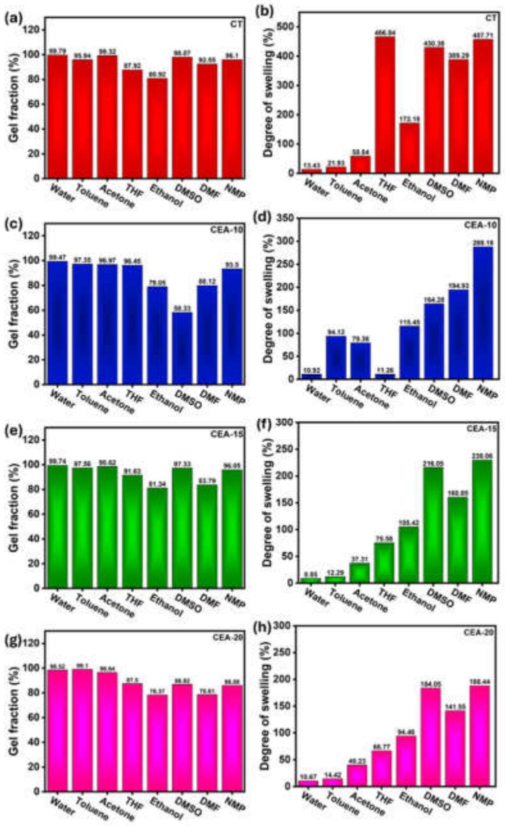

Figure S3. Gel fraction and degree of swelling of (a-b) CT, (c-d) CEA-10, (e-f) CEA-15 and (g-h) CEA-20 samples respectively

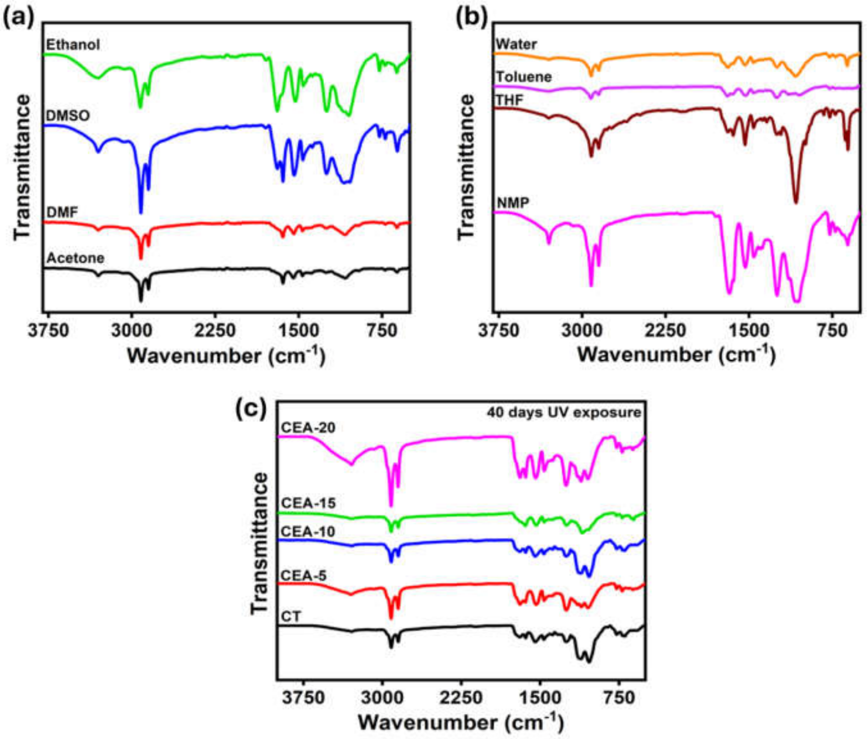

Figure S4. (a-b) FTIR spectra after gel fraction and degree of swelling test of CEA-5 NIPU material (c) FTIR spectra after UV exposure test of all NIPU materials

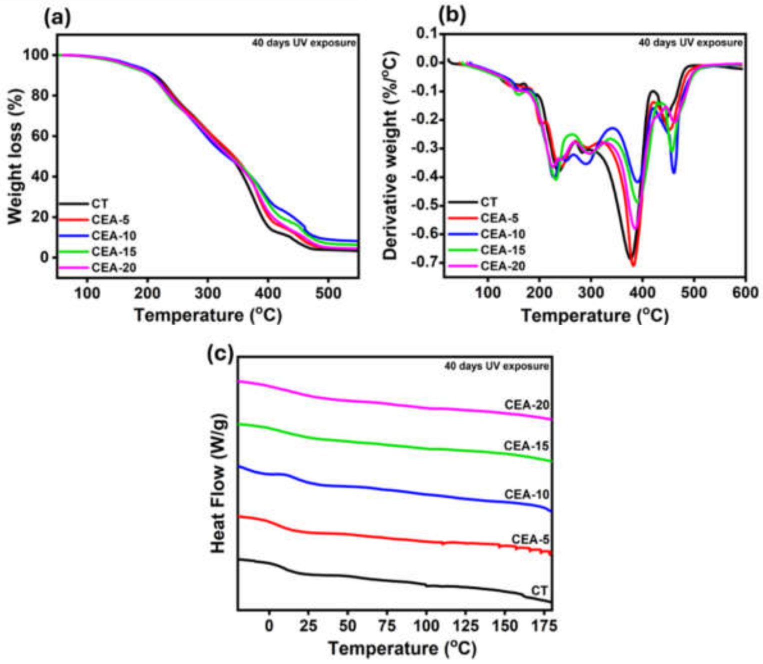

Figure S5. (a) TGA (b) DTGA and (c) DSC spectra of NIPU materials after UV exposure

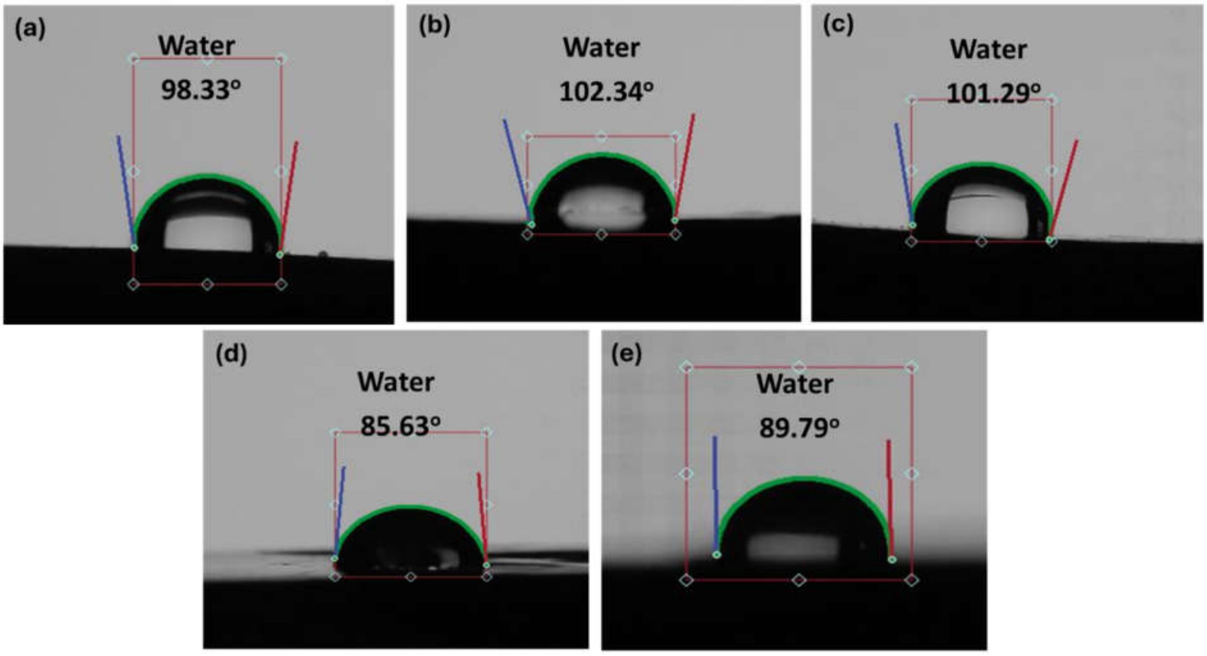

Figure S6. WCA of (a) CT (b) CEA-5 (C) CEA-10 (d) CEA-15 and (e) CEA-20 NIPU materials
